# The Association for Human Pharmacology in the Pharmaceutical Industry London Meeting October 2019: Impending Change, Innovation, and Future Challenges

**DOI:** 10.3389/fphar.2020.580560

**Published:** 2020-11-19

**Authors:** Shahzadgai Khan, Muna Albayaty, James Bush, Joseph Cheriyan, Anthea Cromie, Annelize Koch, Michael Hammond, Stuart Mair, Ulrike Lorch, Steffan Stringer, Jorg Taubel, Timothy C. Hardman

**Affiliations:** ^1^ Niche Science & Technology Ltd., Richmond, United Kingdom; ^2^ Parexel International, Northwick Park Hospital, London, United Kingdom; ^3^ Covance Clinical Research Unit Ltd., Leeds, United Kingdom; ^4^ Cambridge University Hospitals NHS Foundation Trust, Cambridge, United Kingdom; ^5^ Celerion, Belfast, United Kingdom; ^6^ Simbec-Orion Group, Slough, United Kingdom; ^7^ Clinical Quality Management Solutions, Oxford, United Kingdom; ^8^ Quotient Sciences, Nottingham, United Kingdom; ^9^ Richmond Pharmacology Ltd., London, United Kingdom; ^10^ Alwyn Consulting, Guildford, United Kingdom

**Keywords:** early phase clinical development, meeting report, Association for Human Pharmacology in the Pharmaceutical Industry, innovation

## Abstract

The Association for Human Pharmacology in the Pharmaceutical Industry (AHPPI) annual meeting focused on impending change, innovation, and future challenges facing early phase drug development as we move into the second decade of the 21th century. The meeting opened with discussion around the technical revolution in pharmaceutical medicine over the 4 decades since the AHPPI was founded and how transformative technologies have accompanied the introduction of processes such as physiologically based pharmacokinetic modeling. During the meeting examples were presented of how in terms of the development of new therapies, the classic phases of clinical drug development are becoming a thing of the past and the lines between the phases have begun to blur, particularly in the field of oncology. The contribution that monoclonal antibodies have made to medicine and the next chapter in their design and use was also discussed. A representative of the UK’s Medicine and Healthcare Products Regulatory Agency discussed the increasing numbers of requests to approve complex innovative design trials, how novel trial designs are impacting on the traditional linear “phase” approach to drug development and the common pitfalls associated with them. Guidance was provided from a regulator’s viewpoint on what was meant by the term “novel design” and how to submit successful trial applications for such complex trials. In an Oxford-style debate, the audience discussed the motion that “there is no longer a need to include placebo subjects in early clinical trials.” The keynote speaker focused on delivering change in complex environments such as the field of drug development. The afternoon session included presentations on the challenges associated with drug product design, the complexities within non-oral dosage forms and proposed new methods of formulations for drug delivery. Presentations were also given on advances in mechanistic and computational pharmacokinetic modeling and how they have proved to be valuable tools to rationalize and facilitate the process of drug development.

## Introduction

Founded in 1988, the Association for Human Pharmacology in the Pharmaceutical Industry (AHPPI; www.ahppi.org.uk) provides a forum for discussion regarding practical and regulatory aspects of early clinical development of new medicines and continuing education in clinical pharmacology. The AHPPI’s annual meeting held in London on October 11, 2019 focused on the impending changes, innovation and future challenges of early phase drug development and strategies that might be employed by the pharmaceutical industry to address them. By bringing together stakeholders from a range of disciplines including drug development, data intelligence platforms, research organizations, government science policy and clinical trials regulation, the AHPPI committee created an opportunity to share engaging, comprehensive and balanced viewpoints from a broad range of professionals within the pharmaceutical industry. This report summarizes the key observations from the meeting.

## Morning Session

The meeting was opened by the AHPPI Chairman, Dr. Tim Hardman who briefly discussed the technical revolution in pharmaceutical medicine over the 4 decades since the AHPPI was founded. He described how we have seen a multitude of changes ranging from the introduction of complex new study designs to new parameters such as genomics, transcriptomics, and proteomics. The development of transformative technologies has been accompanied by novel investigative processes such as physiologically based pharmacokinetic modeling (PBPK). These modifications have come as a series of “bolt-on’s” that challenge the established clinical framework described within the Good Clinical Practice and International Council for Harmonisation. Dr. Hardman spoke about five key areas he believes are most likely to sit at the heart of future clinical trials: our understanding of disease; the appearance of novel mechanisms of action; the efficiency of the drug development process; reimbursement for new medicines; and ownership of data and the analytical methods used to interrogate it.

After welcoming the conference delegates, Dr. Hardman introduced Professor Emma Baker from St. Georges University, London who chaired the morning session. Professor Baker spoke about a growing skills gap within the pharmaceutical industry and how the newly introduced BSc in clinical pharmacology launched by St. Georges University, London was designed to partly address this challenge. The BSc course places emphasis on providing young graduates with a working understanding around drug development, which is expected to facilitate their introduction into the pharmaceutical industry and reduce the time it takes for them to make useful contributions. Professor Baker expressed how the training course was attracting students from less traditional backgrounds who were applying their knowledge most effectively in this course.

### Phase I Trials in Patients With Cancer a Vision of 2020 and Beyond: Professor Duncan Jodrell (Director, Cambridge Cancer Trials Centre, United Kingdom)

Professor Jodrell described how, as an integral part of the Cancer Research UK Cambridge Centre (CRUK), the CCTC was supporting and delivering clinical and translational research. The goal of the CRUK Cambridge Centre is to improve patient outcomes through application of its research findings and to conduct a wide range of clinical trials in people at risk of cancer or with established early or late stage cancers, primarily at Cambridge University Hospitals NHS Foundation Trust. With over 250 patient referrals annually seeking inclusion in early phase clinical trials from across the East of England, the CCTC has a comprehensive understanding of the challenges associated with delivering complex clinical research in different disease populations, evaluating novel research approaches.

In terms of the development of new therapies, the classic phases of clinical drug development are becoming a thing of the past; the lines between the phases have begun to blur. The advantages and challenges associated with the inclusion of patients in Phase I trials and the building of a science-driven portfolio of early phase trials at Cambridge were discussed.

A specific focus has been assessment of combination strategies, achieved in part by evaluating preclinical growth inhibition data in various cancer cell lines. Establishing an understanding of the combination data generated by these studies is not straightforward. In a majority of cases, interpretation of dose response data requires appropriate mathematical models and the software to implement them. Existing models proved limited and members of the Jodrell group developed the COMBENEFIT software tool (Cancer Research UK Cambridge Institute, 2019).

In oncology, first in human studies are performed in patients, in contrast to many other disciplines. Advantages associated with involving patients in Phase I cancer trials are the early inclusion of a relevant target population, the option to directly monitor for improvements in quality of life and the prompt identification of anti-tumor activity. Challenges associated with the inclusion of patients in Phase I trials result from the confounding effects of underlying pathology and previous treatment such as impaired renal or hepatic function.

There was discussion over how success in identifying novel therapies often depends on investigating orphan drugs in varying combinations tested in small, molecularly defined patient populations responding to the diverse (almost unique) nature of the disease. Drug combinations are frequently employed in patients with cancer. He described how mathematical modeling and an understanding of biological pathways have been useful in identifying the key nodes to target in signaling networks that are involved in cancers. Inclusion of a single biomarker in a basket trial for multiple tumor types can provide untold insights.

The Jodrell group has undertaken collaborative research with AstraZeneca to identify combination partners for use with gemcitabine, an agent frequently used in patients with pancreatic cancer and other tumor types. Using the COMBENEFIT software, gemcitabine was indicated as being a highly synergistic agent with drugs targeting the DNA Damage Response (DDR), in both cell lines and also demonstrating tumor shrinkage in genetically engineered mouse models.

Conventional methods to calculate dosage levels of new and combination drugs have tended to be based on their maximum tolerated dose (MTD). However, simply combining two agents at their MTD may not prove to be optimal in terms of their synergistic interaction or tolerability profile. Professor Jodrell suggested that it is possible to achieve a more thorough interrogation of the interaction of two agents, using model-based approaches to describe an “interaction surface” for the two agents, as described from *in vitro* experiments investigated with the COMBENEFIT software.

It was noted that the debate remains as to whether it is preferable to test the biology of a tumor before initiating treatment with “recommended” doses. To address these challenges, the CCTC is leading a collaboration with AstraZeneca to standardize methods of tumor testing by employing a central review board that makes suggestions for appropriate clinical trials and patient inclusion.

The presentation was concluded by introducing the concept of promoting dose expansion in patients using a dual agent dose escalation strategy to establish combination toxicity profiles and how a solution would be to enroll patients across subsequent cohorts with gemcitabine as a potential sensitizer, to aid modification of the doses for both drugs.

### Dr. Phil Barrington (TranScrip Partners LLP, United Kingdom): Monoclonal Antibodies—Predicting the Next Chapter

The presentation began with an introduction to monoclonal antibodies providing a brief history of their development. Dr. Barrington summarized key milestones from their history: the first “indirect” use of antibody therapy with cowpox immunization against small pox by Jenner in 1796, through the development of “anti-venoms” in the 19th century that came into common use in the 1950s. A key milestone was the identification of a method to generate large amounts of antibodies (Milstein and Köhler in 1975). He discussed how since the introduction of the first monoclonal antibody for clinical use and the means to construct human monoclonal antibodies in the mid-1980s, they have become an important role in the treatment of a broad range of conditions, many of which previously had no clinical solution. Their involvement in medicine has been growing exponentially and in a period of 3 years from 2016 to 2018, 27 new antibodies were approved for clinical use by the US Food and Drug Association (over 20% of all FDA approval that year). He also noted that they have become commercially important making up seven out of the top 10 selling products in 2018.

In considering the next stage of development for monoclonal antibodies, attention was drawn to different structural forms of antibodies and what we have learned from species differences, placing emphasis on what has been learned of camelid and shark biology. Our knowledge of antibody biology has expanded the field to include heavy chain only and nanobody molecules, stereospecific and catalytic monoclonals as well as check point agonist monoclonal antibodies and intrabodies.

The concept of nanobodies as the current “new kid on the block” in terms of their clinical potential. In contrast to conventional monoclonals, nanobodies have low molecular weights and offer the potential to cross the blood brain barrier. They also have better solubility profiles, tissue penetration, and stability. Additional benefits include the ready availability of alternate “starting” parent molecules in yeast libraries, their ability to undergo conjugation (making them useful for new approaches to “imaging”) and possible lower immunogenicity profile.

Caplacizumab was the first nanobody to be approved by the FDA in 2019 and is used to treat the life-threatening autoimmune disease, acquired thrombotic thrombocytopenic purpura. Caplacizumab targets the von Willebrand factor protein that mediates platelet adhesion and inhibits the interaction between the von Willebrand factor protein and platelets. A potential limitation with caplacizumab is one that limits the utility of all candidate nanobodies, a relatively short half-life as a consequence of their high renal clearance. This means daily administration of the nanobody is required in an era when treatments are being selected to have longer periods between doses.

It was noted that most conventional monoclonal antibodies exhibit linear epitope recognition and that stereospecific moieties that recognize three dimensional molecular configurations have the potential to introduce novel opportunities. For example, incorporating stereospecific recognition provides bispecific monoclonals with the potential to detect membranous antigens on cancer cells, which are not readily accessible with conventional linear-specific monoclonal antibodies. Attention was drawn to ecto-S-nucleotidase as a promising immuno-oncology target. The example of MEDI9447 was used to demonstrate how it is possible to combine two different attacking “mechanisms” in one candidate dimer, those of crosslinking and steric blocking. Another opportunity discussed was catalytic antibodies, which not only “recognize” their target antigen but also facilitate its degradation. The audience generally agreed that catalytic antibodies represent a potentially powerful new class of antibodies.

There was discussion over the expansions in topical administration through use of Fabs and nanobodies have been investigated for the treatment of skin diseases such as psoriasis and atopic dermatitis.

Dr. Barrington described the mechanism of action behind bispecific monoclonal antibodies. To create a bispecific antibody, the Fab can be cut, which effectively splits the antibody into two halves. As the Fab is comprised of both heavy and light chains, in order to stop the Fab from falling apart, a “linker” molecule is used to produce a “single chain variant” and these can be “added” onto the structure of an antibody. An alternative method is the “knob in hole” technique where a pocket is created on one end with a protrusion on the other, which allows the heavy chain to align correctly. Potential issues with bispecific antibodies are that they tend to become “fixed dose” combination products, which are not generally favored by physicians. There may also be issues with the parent molecule construction. The obvious approach would be to have a 1:1 ratio for your specific apparatus, and yet it is possible to select any ratio—whichever gives the best results. Theoretical target binding shows that 2:1 or 3:1 ratios are optional. The frequency of targeting binding is unpredictable and is dependent on the molecular weight of the antibody format. There is also a question as to whether bispecific antibodies are more immunogenic and this will need to be thoroughly assessed before they can be adopted widely.

Potential methods of oral delivery of peptides and proteins through the selective use of lipids, formulation for lacteal absorption, paracellular transport, with “tight junction” openers, promotion of diffusion via the mucus layer in conjunction with nano-objects that may facilitate diffusion via transcellular pathways, permeation enhancers and gastric injection of proteins were described. However, issues generally associated with these methods include; their requirement to be administered after fasting; possible damage to the gastrointestinal tract; and reliability of their delivery in a setting where bioavailability is unpredictable challenges regulatory approval requirement. Alternative expression systems may enhance such approaches through the co-opting of genetic modification techniques. New methods of genome editing are expected to become available in the near future for transgenic animal expression systems where proteins can, for example, be expressed in milk.

In revisiting the high molecular weight limitation associated with monoclonal antibody that restricts their tissue and cellular penetration, the concept of intrabodies where “antibodies meet gene therapy was introduced.” Intrabodies are antibodies that are expressed intracellularly and remain within the cell cytosol or endoplasmic reticulum. To produce intrabodies, DNA is introduced into the cell by retroviral delivery systems often used in gene therapy. This technique allows highly specific targeting of intracellular proteins. It is postulated that this approach could reduce the potential of unwanted adverse effects associated with systemic exposure to antibody therapies, although no such therapeutic approach has yet been tested in humans. Nevertheless, *in vitro* and *in vivo* studies in a variety of animal models have shown promising activity.

The potential for checkpoint antagonist and agonists was also discussed briefly in closing the presentation, giving examples of molecules in development as well as the opportunities when targets are combined in bispecific molecules.

### Challenges of Novel Trial Designs on Clinical Application Approval: Dr. Kirsty Wydenbach (Medicine and Healthcare Products Regulatory Agency, United Kingdom)

The Medicine and Healthcare Products Regulatory Agency (MHRA) are seeing more and more drug development pathways employing clinical trials that address multiple clinical questions in a single application and which may be termed “complex innovative design trials.” Dr. Wydenbach spoke about how novel trial designs are impacting on the traditional linear “phase” approach to drug development and the common pitfalls associated with them during the initial stages of regulatory approval. Guidance was provided from a regulator’s viewpoint on what was meant by the term “novel design” and how to submit successful trial applications for such complex clinical trials.

It was acknowledged that the MHRA may previously have been considered overly cautious in its approach to clinical trial applications that have attempted to employ less traditional study designs. However, they have come to recognize the benefits that can be achieved by adopting a flexible approach to the way clinical trials are conducted. They have been actively tracking all novel designs being requested. In the future, although the application, review and approval methods will be the same regardless of the trial design, they expect to take a more proactive approach to innovative ways to develop new medicines. The agency plans to share more of the metrics they collect on the trials they approve and provide more guidance to sponsors on how to get their trial approved, acknowledging the level of guidance that has already been achieved by the US Food and Drug Administration. Feedback from organizations working with the MHRA suggests that sharing its information on the design and implementation of trials will be beneficial to the industry as a whole. In response to this feedback, a group of stakeholders from organizations within the pharmaceutical industry have been developing a consensus paper that will cover all aspects of trial design and will bring to light key training and education requirements.

A “recommendation paper” on the initiation and conduct of complex clinical trials developed by the Clinical Trial Facilitation Group, a European regulatory forum spanning all member states of the EU, was published in February 2019 (European Clinical Research Infrastructure Network, 2019). It is hoped that the guidance provided will increase harmonization and reduce the number of trial applications that are rejected by agencies including the MHRA. Master protocols are frequently associated with basket, umbrella and platform trial designs where the core protocol plus sub-protocols can be submitted for approval either as individual clinical trials or as part of a single complex clinical trial. It is generally agreed that master protocols must describe the overall clinical trial design including components and operational aspects applicable to any related sub-protocols (such as the rationale behind the trial objectives, endpoints, and risk-benefit assessments), shared procedures relating to safety monitoring and reporting as well as subject screening, eligibility, and/or treatment allocation.

The traditional phases of clinical trials were described as becoming somewhat out-dated and the emergence of seamless development in the form of trials that encapsulate aspects of several phases of development within single studies. When incorporated appropriately into clinical programs, whether they are basket, umbrella, matrix, or platform trials, they offer the potential for accelerated development reducing the time normally taken for researchers to get access to data generated by individual studies. However, a number of pitfalls are commonly associated with clinical trial applications for these novel types of trials that have tended to impact on whether or not they gain regulatory approval. Sponsors working with complex trials often overlook the possibility that changes to study conduct during the course of implementing adaptive modifications can have an impact on the primary objective of a trial, so that it no longer aligns with the original hypothesis. Frequently there is no clearly stated “end” to a study. Sponsors also often make modifications to their protocols without properly qualifying the reasons for such changes in the form of a formal rational. This tends to serve as a red flag to regulatory agencies, raising questions. Often, the lack of clear explanations behind changes results in the delay of an application, or even rejection in those cases where sponsors fail to provide adequate justifications.

The MHRA has adopted a pragmatic approach when considering clinical trials applications employing novel designs. In providing her “top tips” to ensure the success initial drug trial applications, she advised that Sponsors clearly justify their choice of trial design, the investigations and their use of investigational medicinal products, where relevant providing their reasoning behind decisions to employ adaptive designs over a more traditional approach (Medicines and Health Regulatory Agency, 2019). Criteria for closing or expanding a study group should be included in the initial application if the Sponsor feels there is the slightest possibility of this happening. It was noted that the MHRA require scientific reasoning for the introduction of changes and will rarely find logistical benefits alone acceptable—an “easier” design is not sufficient explanation to justify a change to your initial trial application. A list of potential adaptations that the Sponsor may implement must be included at the outset. For example, if a Sponsor considers that they may include an additional study cohort, then they are more likely to achieve a positive response and face less resistance from the agency if they introduce this possibility as early as possible.

Dr. Wydenbach discussed how, when submitting a substantial amendment to complex protocols, a narrative description of changes does not necessarily provide an easy way for assessors to make a comparison between original and amended documents and advised that it can be better to use tables as a way of presenting modifications. She indicated that Sponsors should put more effort into describing the thought processes behind any modifications. It was highlighted that for amendment submissions there are no options for the agency to respond by questioning the Sponsor’s approach and if the agency is left in any doubt over the acceptability of a modification, it has no option other than to reject the submission. It was also advised that Sponsors ensure that their control groups remain valid throughout the trial as the requirements or purpose of such a group often shifts when changes are made to the treatment pathway. If changes are made to what happens in the control group, particularly where a control group is common to several arms in a trial, then benefits, risks and safety aspects relating to this group also need to be reassessed. A clear explanation should also be provided as to why any new, additional arms were felt to be appropriate as part of an amendment rather than completing the current study and introducing a new trial.

The presentation was concluded by describing how in the future the MHRA will actively promote the use of complex and innovative trials, taking a more proactive stance and offering greater assistance with the implementation of novel protocol designs. The MHRA are working toward maintaining the UK as a competitive space for the conduct of clinical trials in partnership with Sponsors and CROs. To achieve this, it is currently developing a novel trials implementation plan which will focus on engagement with stakeholders, feedback from workshops, internal training, providing guidance, and collaborative engagement with organizations such as the National Institute for Health Research and the National Institute for Healthcare and Care Excellence. The importance of communication between the Sponsors and the MHRA in delivering successful trial applications and stated “ask for advice” was stressed. However, she added that Sponsors should not be surprised by significant questions at the time of an application if they do not follow the guidance provided to them.

### Value of Placebo in Phase I Studies; an Oxford Style Debate: Dr. Peter Dewland (MAC, United Kingdom) and Dr. Sven van Dijjkman

A great majority of early phase clinical studies follow a blinded, placebo-controlled design, often involving a small number (six to eight) of subjects receiving doses of active treatment and one or two subjects being given placebo. Studies usually investigate multiple dose levels, each mirroring the same placebo-controlled design. It is the generally held belief that pooling placebo data from each dosing cohort (typically somewhere near a number equal to the number of active treatment subjects included in a single dose level) provides a cohort that can be used to assess the actions of the “active” therapy. As such, randomized, double-blind placebo-controlled trials have come to be considered the “gold standard” approach to clinical study design for producing data untainted by bias. However, beyond anecdotal opinion, there are very few published evaluations that provide empirical data to support the ready acceptance that such study designs are necessary to provide high quality data, while evidence suggests that the method itself may be flawed. In an Oxford Style debate, Dr. Sven van Dijkman defended the motion that “there is no longer a need to include placebo subjects in early clinical trials.” Dr. Peter Dewland (MAC, United Kingdom) countered the motion and the session was chaired by Dr. Emma Baker (St. Georges University, London, United Kingdom). A poll of the audiences’ opinion on the motion was performed prior to the proceedings. Only one member of the audience felt that the proposed concept was acceptable and placebo subjects were unnecessary.

Dr. Sven van Dijkman opened his presentation by questioning whether the motion represented a human or medical perspective. He reasoned that although factors such as pharmacokinetics, pharmacodynamics, tolerability, and safety need to be quantified and appropriate dosages determined during early development, the use of placebo administered subjects in Phase I studies does not provide useful insight into an agent’s toxicity, safety, or tolerability. There is some skepticism as to whether data of any real value can be obtained by comparing the behavior of new molecules in healthy subjects with those given placebo. He reasoned that it could easily be argued that if any comparison is necessary then placebo data generated during a trial could be replaced with historical data from previous studies. Comparator data could also be used from pre-dose samples, time-matched baseline assessments that are often conducted for such studies or past data from placebo subjects included in similar studies. This would mean that healthy subjects avoid the exposure of any risks associated with placebo and procedures during participation in a clinical study, while also reducing the cost, complexity, and time taken to run early phase trials.

Despite the lack of scientific support, Dr. Dijkman stated that placebo drugs are usually administered in Phase I trials to prove that “snake oil” is not being sold, and yet, he noted the small number of placebo subjects typically included would not be sufficient to determine the unknown effects with any level of confidence. Most serious adverse events are rare and their signal cannot be detected in small groups thus a placebo group would be unlikely to provide beneficial data or insights into the Phase I setting. Furthermore, the use of placebo is associated with “highly variable outcomes,” making it difficult to establish early signs of risk and efficacy by comparing the active drug against the placebo. Psychosocial and cultural factors may indirectly modulate observations with the “inert pill” which, in the end, fail to produce randomized effects.

A case of a study was related that was conducted with levodopa in patients suffering from Parkinson’s disease. The study observed an increase in placebo effects as the likelihood of subjects receiving the active drug increased. Issues were raised of two specific cases where study designs included placebo groups, but failed to predict any risks, namely TeGenero, the German biotechnology company whose experimental drug, TGN1412, caused the hospitalization of six men and the Bial trial, for experimental drug BIA10-2474 resulted in the death of a study subject. He concluded that in both cases a clearer understanding of the pharmacology was what was missing and that the availability of placebo data could not have prevented such serious outcomes.

A structured modeling approach was proposed to determine the detection of placebo effect against a dose-response curve. Observations in difference in subject response might be observed if historical data was combined with disease models. There are studies that provide data that predict the placebo response differentiated from the actual treatment effect and show the subjects’ profiles change over time with some diseases. For example, bipolar disorder studies have shown a clear “placebo effect” over time in large numbers of patients, from combined data across 11 clinical trials and five different drugs. Similarly in depression, random fluctuations in bio-behavioral health were observed in data on 700 patients from six clinical trials.

The final point was that information given to the subjects in the trial can determine the size and variability of any placebo effect. Phase I trials should be given careful consideration to the inclusion of placebo subjects as they fail to contribute to predicting serious adverse events or identify clinically relevant actions of the active compound. The next critical step suggested in first time in human trials is a shift in paradigm to improving efficiency of trials through structural modeling instead of recruiting volunteers. This has the potential to reduce costs and the burden on trial subjects. Overall, an underlying exposure-response relationship needs to be established.

Dr. Dewland’s motion described the fundamental rationale behind Phase I trials. He agreed that the general adoption of placebo-controlled trials had been based on theoretical reasoning and intuitive attractiveness rather than a compelling body of data. He noted that attempts to investigate the benefits of placebo in clinical trials systematically have been relatively scarce. However, he referred to the Integrated Research Application System (IRAS) form, stating the current standards of Phase I trials which are considered to be a less subjective approach and are conducted to determine the safety, dose levels of response and first time in human effect of an investigational medicinal product. A standardized model approach to placebo data was argued as not feasible in Phase I trials as all treatments are varied and differ in mode of action, e.g., biologicals, siRNA, and small molecules. Equally, it was argued that all humans are different physiologically thus no two clinical trial populations could possibly be identical.

It was noted that in the studies presented by Dr. Dijkman in support of a modeling approach data sharing were mostly conducted in studies into depression that were larger than typical Phase I studies and in most of these cases the placebo groups were required to differentiate between the effects of the investigational agents. In contrast, Phase I trials are focused on establishing safety, so the aim to test for effectiveness in a study may not be justified. The mathematical modeling approach proposed by Dr. Dijkman’s was challenged as being based around the “symptomology of the disease” rather than focusing on understanding the investigational medicinal product. Subsequently, the use of historical data and disease models was considered unsuitable for Phase I as the model is specific. Dr. Dewland concluded with the quote, “if it ain’t broke, why fix it?” Placebo cohorts are, he argued to provide better reflective conditions of the trial and active drug.

The audience quizzed both candidates on different aspects of their presentations. Much of the focus was around establishing causality of safety episodes in the absence of placebo group with focus on aspects such as more severe episodes of liver toxicity. Other discussions focused around unbiased interpretation of mild effects without placebo data or observations for comparison and whether interpretation of data from placebo-free studies would be susceptible to subjective factors. A suggestion made was that inclusion of placebo group may contribute to higher statistical power through accumulation of more data.

Following the lively discussion, the audience were asked once again to vote on the two positions. Following the presentations, 14 members (approximately 20%) of the audience had changed their position from “against” to “for” the motion.

## Afternoon Session

### Keynote Address: Dr. Stephen Carver (Senior Lecturer in Project and Program Management, Cranfield School of Management, United Kingdom)

In line with the theme of the conference, Dr. Carver gave an insightful presentation on delivering successful change. He first noted that many long-established companies, even entire industries, are being disrupted by the challenge of change. He observed that the pharmaceutical industry is a business with its foundation and profitability firmly rooted in being able to deliver change, relying on a capacity to disrupt, innovate and regenerate while constantly being challenged by innate forces that resist change. These forces not only reside in the government agencies that manage and regulate the industry but are inherent to the culture that pervades it. Topically, he referred to Darwin pointing out that he was not responsible for the commonly guided phrase “survival of the fittest.” What Darwin actually said was “it is not the strongest of the species that survives nor the most intelligent. It is those that are the most responsive and adaptable to change.”

The concept of VUCA was introduced that describes situational volatility, uncertainty, complexity and ambiguity and originated from the US Army War College in the 1980s, on the end of the Cold War. Change is a common thread that runs through all businesses irrespective of their size, industry, or age. Our world is changing rapidly and the science that drives the pharmaceutical industry is dictating that organizations must also change quickly. He concluded that those industry players who address change will thrive while those that do not will struggle. Many organizations are only equipped with outdated models of change management, too often reflecting a traditional style of management ill-suited to a VUCA world.

The presentation summarized the various different types and drivers of change and the approaches we often see being adopted in attempts to address them in the context of making a journey by plane. He described how, when planning a journey, we overlook many of the risks associated with air travel and how it reflects our expectations with regards to project management. He also pointed out how over 50% of change projects fail and asked the audience whether they would consider flying if 50% of flights failed. It was highlighted how it is possible to define change in project management terms as the discipline of paying meticulous attention to every aspect of a project or program and how this was just simple common sense. However, as Voltaire remarked, “the only problem with common sense is that it’s not very common.” In reviewing the characteristics of organizations well-adopted to change, Dr. Carver listed: a work-base consisting of people who thrive on change; have open channels of communication and feedback; and a flexible work force.

It was pointed out that freeing people up to generate and develop great ideas is not the same as letting amateurism run amok. Indeed, one of the key reasons many projects fail comes down to insufficient focus on the development and training of great project and change managers, with opportunities for advancement often being given to the ill-equipped. Rigorous training and accumulation of experience greatly improves the ability of leaders to cope with the stresses and strains of designing and steering change.

These points were illustrated with the dramatic example of calm decision-making by Captain Chesley Burnet Sullenberger III when landing his stricken US Airways Airbus in the Hudson River in 2009. Captain Sullenberger employed his rigorous training in a crisis, managing huge complexity, communicating clearly, and knowing what rules to bend in order to achieve a successful outcome for all stakeholders.

Replaying the voice recording taken during the incident, Dr. Carver highlighted how quickly things went wrong but also how all participants remained calm and trusted their training. As with all disaster scenarios, there were checklists and protocols for those involved to follow, but in this case, Captain Sullenberger jumped protocols and used his own judgment. He was equipped to handle such situation because of previous training he had undergone in flight simulators.

Dr. Carver introduced the term “complex and complicated” while describing the daily pattern of air traffic control over Europe. He discussed how “complicated” refers to systems that are linear and predictable simply requiring diligence and effort to master. He noted that systems like air traffic control can simply be described as complicated. In contrast, complex systems are non-linear and unpredictable requiring experience to understand and agility to negotiate (Maylor et al., 2008). There are three dimensions of complexity: structural, emergent, and socio-political.

Tackling these dimensions can be difficult and requires different skill sets. Structural complexity would depend on the number of factors that need to be considered—patients, people sites, etc. This is something the pharma industry is good at. Emerging complexity (the degree to which the world around you is changing what might be termed- “the unknowable”) requires the investment of time to understand what is changing, for example the emergence of new technologies. Socio-political complexity requires an understanding of how to address a wide variety of stakeholder issues and relationships; these might be termed “political” skills. Traditionally, the mastery of political skills has not been people development priorities in organizations involved in the pharmaceutical industry. However, it is apparent that we need to rethink how we prepare leaders and project managers for the challenge they are facing. The flying analogy was used to discuss the concept of managing simulations in the manner of fighter pilots where the situation is constantly changing. Good managers tend possess two of the three complexities and so you should alter the make-up of your team to ensure you cover all potential complexities.

He concluded his presentation by highlighting how research has indicated that the one complexity most people find difficult to manage is the socio-political aspect. And yet, it was generally agreed within the audience that the area that most clinical scientists focus on is structural complexity. Dr. Carver closed stating that if there was a single reason why most trials fail, this would be it.

### The Biopharmaceutical Impact of the Delivery of Molecular Drugs via Non-Oral Routes of Administration: Dr. Eddie French Formulation Consultant, TEKH Consulting Ltd.

Dr. French introduced the challenges associated with drug product design, the complexities within non-oral dosage forms and proposed new methods of formulations for drug delivery. He noted how fundamental questions are usually raised during the manufacturing of a drug around issues of dosage, route of administration, pharmacokinetic profile and mechanism of action as well as the intended target population. It is generally considered the responsibility of pharmaceutical scientists to convert the active ingredient of a pharmaceutical product into an easily administered dosage form that can be used to deliver the correct amount of a drug to its proposed site of action. Often the product itself is presented as raw compound (in a vial), with the assumption that the issue of formulation is no more challenging than simple encapsulation or incorporation in a tablet form. However, getting the formulation right is a complicated, involving a plethora of technical hurdles and requires thorough understanding of both the molecular characteristics of the active ingredient and the biology of the target.

The pharmaceutical suitability of drug candidates is triaged early during drug development and tends to be based on computer modeling, high-throughput screening and cell-based assays that predict pharmacologic activity. It is, however, much more difficult to predict factors such as drug absorption, distribution, metabolism, and excretion (ADME), which typically require evaluation and understanding in both the *in vitro* and *in vivo* settings. The final product may be adopted into any of a selection of delivery mechanisms: IV, intramuscular (IM), intranasal (IN), or intradermal (ID)/transdermal administration. However, oral administration is still the first line and preferred route of drug delivery. Other routes, such as ocular or inhaled delivery, have also been developed for localized, site-specific drug administration. Each route of administration faces specific barriers against delivery.

Historically (and currently), the standard and most well-defined route of administration is via oral delivery, where our understanding has been augmented by mathematical modeling which has been shown to successfully predict the absorption, distribution, metabolism, and excretion of oral drugs. Oral drugs can be easier to assess using precedented *in vitro* models that can employ bio relevant media and well-mapped animal models. Although some drugs have local effects in the gut, most enter the systemic circulation to act in other parts of the body. As a route for delivery the GI tract can be divided into upper and lower parts. The structure of the GI tract is similar in all segments. The characteristics and physiology in the small intestine provide good opportunities for drug absorption which is relatively well understood as such, the predictive modeling of orally delivered formulations is reasonably mature with established simulations and animal models.

Although oral delivery remains the most prevalent, other routes of administration are widely used, many drugs require local delivery, e.g., for drugs that cannot be administered orally. For example, ophthalmic drugs tend to be applied directly to the eye by drops to increase targeting efficiency and minimize systemic expose. Similarly, inhalation is the preferred mode of delivery for local treatment of the lung, which also avoids potential systemic side effects. It can be used for drugs needing rapid onset enabled by quick absorption and in addition this mode of administration has potential for drugs affected by first-pass metabolism and for macromolecular drugs that would otherwise need to be injected. Transdermal delivery is sometimes considered for drugs that undergo significant or variable first-pass hepatic metabolism or drugs that would benefit from steady plasma concentrations enabled by a controlled release in the form of a patch, but is very dependent on and limited by the physiochemical properties of the drug. The global market for non-oral drugs is expected to double from its current value of about $45 billion within the 5 years.

Dr. French noted that generally for injectable drug candidates early clinical work is conducted using IV administration for the sake of speed and convenience, but many drugs may later move to alternative injectable routes of administration that are envisaged to be the way final product is given to patients, e.g., subcutaneous or intramuscular injection (IM). This approach has been relatively successful; however, bridging between the dosage forms can be complex as *in vitro* experiments don’t give a true picture of delivery to a human and many animal models can similarly be non-representative. For example, the dosing levels between an IV and subcutaneous (SC) route are not interchangeable and require different formulations and/or volumes. Equally, there is a misconception that administration by SC or IM gives 100% bioavailability but this is not the case. When assessing a SC delivery system using animal studies to predict bioavailability there is a high level of species and molecule dependency that needs to be taken into account. The ADME will also depend on the characteristics of the drug for example, with higher molecular weight molecules (greater than about 16 kDa) being distributed from the injection site via the lymphatic system whereas smaller molecules will distribute by lymph and directly to the capillaries. Dr. French went on to describe the impact the skin’s adipose tissue (body mass index and gender dependent) and connective tissue (age) along with depth of needle penetration depth impacts on dose.

How biopharmaceuticals behave immediately after SC administration is still not fully understood. Factors that influence the percentage bioavailability for SC delivery such as concentration, pH, lipophilicity, protein binding and metabolism directly influence the release from the injection site. Tools are becoming available to help map this behavior. The “Scissor” technology was developed by Pion Instruments in conjunction with the University of Bath. Scissor is an instrument that can simulate the stress conditions and environmental transitions that a biopharmaceutical drug experiences when injected subcutaneously. The technique has been used for large macromolecule and peptide sand could be could be extended to the application of small molecules. It was expressed that the use of such instruments provide a good foundation for understanding the factors that influence delivery via the subcutaneous route and enable more rationale formulation design. Overall, he concluded that the SC route of administration offers a great deal of potential as a route of delivery for many molecules but requires a significant “understanding” of the many variables involved to facilitate rationale formulation design and any bridging between injectable systems.

For the last part of his talk, Dr. French focused on factors he understands to be the key elements which impact on the success of dry powder inhaler drug delivery to the lungs (Tristan et al., 2009). Dry powder inhalers (DPIs) are an important delivery system for many drugs used in respiratory diseases. They serve a good delivery option for various patient populations, and may help to overcome several limitations associated with other types of inhalation delivery systems (e.g., accuracy and reproducibility of the delivered dose, patient compliance and adherence issues, as well as having a better environmental profile). There are more than 20 different dry powder inhalers on the market delivering active pharmaceutical ingredients for local and systemic therapies. Dry powder inhalers demonstrate varying performance levels depending on the mechanism of deagglomeration, aerosolization, dose metering accuracy, and the inter-patient variability. During development, manufacturers focus on improving aspects characteristic of their specific DPI devices, depending on the intended type of application and specific associated requirements.

Inhalation devices are designed to deliver a predefined dose of a drug reproducibly to the central and small airways or alveolar region of the lung. Particles with a mass median aerodynamic diameter of 1–5 μm are effectively deposited; scientists have become adept at modeling and predicting deposition of particles in the lung. However, a broad range of factors affect delivery to the site of absorption and the eventual drug absorption. Using this knowledge and depending on therapeutic need, different drug modalities can be targeted to different areas of the lung. For many respiratory diseases the target area is the central airways. Local treatment is desired as it involves delivery to the target tissue, negating the necessity for high systemic exposure which can be associated with unwanted side effects. The approach is different when the lung is employed as a portal for systemic delivery under such circumstances the target for drug delivery is the deep lung facilitating allows alveolar absorption. Permeability in the alveolar tissue tends to be high, the available surface area is large and enzymatic degradation is low. Consequently, the process of absorption is less limited (molecule dependant) and delivery becomes largely dependent on transport to the alveolar surface, which depends mostly on the delivery system.

Animal models can be useful for evaluating the fate of inhaled materials and providing valuable information that can be explained later in inhalation product development. When selecting the appropriate animal model, many parameters are considered, including lung structure, disease pathology, and for macromolecules the immunological similarity to humans. Although the data obtained from preclinical studies is valuable in advancing inhalation drug delivery, extrapolation to humans is not straightforward due to the differences in the physiological differences across species and the mode of inhalation. For example, rats are obligate nasal breathers primary local impact surfaces for drug being inhaled will differ from larges species and humans.

The use of traditional *in vitro* dissolution techniques for DPI formulations do usually to correlate well with *in vivo* pharmacokinetic data requiring more advanced and well thought through models to help us predict exposure levels in humans. As a comparison dissolution in simulated gastric and intestinal fluids in combination with permeability across Caco-2 monolayers can provide a good prediction of bioavailability for oral formulations. In similar fashion, simulated lung fluids may also be a tool to assess orally inhaled formulations. Many versions of this have been assessed with similar pH and osmolality but different amounts of lipid protein and surfactant have been used and to date a standard has not been fully agreed. The dose level can also impact the selection of the dissolution model as mimicking relative distribution and drug loading at an absorption site can highly influence observed dissolution rates. The local saturation achieved around dissolving particles, which is related to the density of deposition, may cause interference between dissolving individual particles. This would be different to the dissolution from a single agglomerate of drug, but the dose would look same. As such, structure of the deposited particle is also a key factor. This is complicated by the different absorption rates in different areas of the lung and the fact that, as particles can be removed by mucociliary clearance, there is a temporal aspect to studies that needs to be understood. As such, similar to oral absorption the speed of dissolution can highly influence availability for poorly water-soluble drugs.

In closing his presentation, the message was reiterated that the formulation scientist plays a crucial role in determining success of early development programs for both new chemical entities and generic medicines. Work that has been ongoing to produce a better understanding of the biological and physiochemical processes behind inhaled delivery implies that in the future drug candidates for pulmonary delivery should be more scientifically understood and less of a “black box” approach.

### Simulation and *In Silico* Environment in Phase I Trials: Dr. Marco Siccardi, University of Liverpool, United Kingdom

Dr. Siccardi discussed how advances in mechanistic and computational pharmacokinetic modeling have provided valuable tools that can be used to rationalize and facilitate the process of drug development.

Computational pharmacokinetic modeling techniques are frequently used to provide insights into drug disposition and to aid in selecting optimal drug candidates for development, predict human dosing from preclinical pharmacokinetic and pharmacodynamic data and simulate clinical scenarios to optimize patient management. The presentation was opened with reminding the audience that drugs have traditionally been evaluated for their safety and efficacy using randomized control trials. One limitation with this approach is in establishing how such drugs are likely to act in the real-life, clinical setting. The complex nature of human diseases, significant variations between individuals and the variability in the disposition challenges our ability to predict how new agents might affect patients. The solution has been to establish a molecular profile for a drug based on the available laboratory and clinical information.

Through the use of simulation and *in silico* clinical trials, we have introduced the possibility of bridging the gap between the limited information available in the preclinical setting and a broader understanding of a molecule’s performance in early phase clinical development. A specific benefit of *in silico* strategies is their ability to generate large amounts of data from the information provided to; data which can then be used to understand underpinning mechanisms, simulate relevant scenarios, and select suitable candidates.

Insight acquired from these processes can accelerate the development of novel therapies and advanced new materials. The synthesis of data introduces an additional aspect of research that has the potential to streamline the drug development and regulatory process as well as reduce the burden of preclinical testing while also introducing an opportunity to optimize clinical trial designs. Dr. Siccardi proceeded to illustrate the potential of simulation with past examples where modeling was employed to i) identify patient characteristics and ADME processes influencing the efficacy and toxicity of therapy; ii) optimize dosing strategy; iii) streamline the drug development and regulatory process and; iv) develop novel formulations.

In HIV therapy, patient characteristics and their pharmacokinetics to markedly impact on the efficacy and toxicity of medicines. Although a plethora of anti-HIV medications have been proposed over the past 2 decades, the challenge has been identifying the optimal candidates on which to best focus resources. Traditional comparison of clinical performance takes time and can put the lives of test subjects at risk. Computational pharmacokinetic modeling can use actual data in the laboratory to characterize each of the different mechanisms that influence drug disposition. Quantitative structure activity relationship (QSAR) relates to the mathematical relationship between biological activity of a molecular system and its physiochemical and geometric properties that dictate characteristics such as: blood plasma ratio, fraction unbound, plasma stability, hepatic clearance, and renal clearance. Rational evaluation of QSAR models can be used to identify the most promising anti HIV drug candidates. The integration of potency data from large datasets predicts theoretical pharmacokinetics and validates preliminary simulations of theoretical dosing strategies. Furthermore, data harmonization in a computational environment means these models can be used to identify key variables and generate molecular predictions for candidate optimization.

Patient adherence to specified dosing regimens can be a major reason for treatment failure in that they can easily end up with suboptimal therapy. Often, the reason for non-adherence is associated with a dislike for a drugs route of administration for example, many patients find injections unpleasant. The use of Long Acting Extended Release Antiretroviral Resource Program (LEAP) studies were discussed and the use of implanted devices which release anti-retroviral drug over time negating the need for regular injections. He spoke about how the introduction of micro-needle array patches, solid-coated, non-biodegradable materials that penetrate the visible epidermis providing access to the dermis, provide a patient friendly, low cost and minimally invasive and pain-free route of drug delivery. As such, they have the potential to improve patient adherence. Drug release from nano-particulate formulation can act as the rate limiting step impacting on local and systemic delivery. Modeling has been able to provide insight into the performance of existing twice monthly injections versus monthly intradermal patch applications.

Despite the different experimental methods available for characterizing drug-drug interactions, predicting how the various different biological systems responsible for disposition can challenge dose optimization in certain population groups. For example, dose optimization is a particular issue in neonates since their immature ontogeny and ongoing system development can complicate scaling of doses. The use of physiologically based pharmacokinetic modeling was described as a method to predict drug-drug interaction through estimating enzyme modulation. This approach can be used to minimize the potential for interaction and the risk of adverse events and facilitating dose optimization.

In his final example of modeling, the concept of using patient ADME characteristics to estimate potential efficacy and toxicity of drugs. The variety of shapes and sizes of the human body brings challenges when determining drug disposition. For example, functions such as cytochrome enzyme expression, organ blood flows and volume and gastrointestinal physiology vary in neonates, overweight patients and the elderly. Equally, drug disposition may be affected by comorbidities such as renal or hepatic impairment. Our understanding of how the catalytic activity of key enzymes markedly change in the first years of life, such as the different expression of CYP3A4 and CYP3A7 at 5–7 days old and 3+ months, can help us predict the pharmacokinetics profile of certain drugs. Modeling with PBPK has been used to optimize dolutegravir in neonates (Bunglawala et al., 2019). This particular patient group represent a vulnerable population where the combination of a rapidly developing physiology and immature ontogeny can complicate dose scaling. And yet, clinical trials in neonate are difficult to rationalize in terms of risk as it can be challenging to determine the optimal dose to test. In this case, *in silico* predictions informed dosing selection, removing the need for ultra-safe subtherapeutic starting doses, accelerating registration.

The presentation was concluded with stressing the importance of artificial intelligence in acquiring a deeper understanding of pharmacokinetics and optimizing existing therapies. Machine learning is a method of data analysis that automates model building. As a specific branch of artificial intelligence, it assumes that “systems” can learn from the data they are provided with, identify underlying patterns and (eventually) make objective conclusions with minimal human intervention. Artificial intelligence can facilitate the prediction of the magnitude in drug-drug interactions, help design novel formulations and establish efficacious dosing in niche populations. Thus, the identification of dose adjustments can help with upcoming rational designs of future studies, revolutionizing the drug development process.

### Data Driven Transformation in Drug Development: Dr. Satnam Surae, Aigenpulse Platform

Dr. Satnam Surae opened his presentation by describing how evolving technological landscapes are stimulating the prevalence of data science literacy in early phase drug development and how data-driven companies employing integrated and advanced analytics are out performing their competitors outside the pharmaceutical industry. Everyone is aware of the productivity challenges faced by the industry; that spending on research and development is increasing while the return in terms of the number of regulatory approvals of new therapeutic agents has been disappointing. To gain a competitive edge it will be necessary for pharmaceutical companies to embrace the innovations around data science and apply this new-found understanding to drive drug discovery, empowering research, and development and thus ensuring a sustainable future.

Over the last 4 decades there has been substantial growth in the amounts of large and small data accumulated in early phase drug development although there have been many challenges in the accessibility and quality of this data. For example, a broad variety of experimental outputs are generated when constructing the preclinical packaging and yet the result is often poor quality, incomplete data. The data accumulated are often recorded in proprietary formats and protected by security and data permissions and yet these data could provide value beyond registration packages. A shifting landscape in machine, active and cloud-learning data streams as yet limits the transferability of this research data restricting how it may be interrogated.

Web-lab scientists are advised to become more technologically aware and make better decisions based on data-driven ideas. Data bridges and open data standards are being promoted in the research and development environment to better organize research data making it easier to share and exploit. But initiatives are required to better promote research computing and support the rapid deployment of scalable tools, e.g., containerization and cloud deployment. Dr. Surae predicted that code versioning, testing and output validation as well as agile methodologies will become prominent in the industry and encourage better practice.

Bioinformatics and computational biology are required to bridge the gap between wet lab, data science, software engineering, and information technology. Instead of needing to ask permission and manual handling and data movement, computational biologists and bioinformaticians should be able to push and pull data between systems. It can be predicted that companies and/or scientists that fail to adopt advanced data technologies will be superseded and outcompeted by those who do. To resolve this, it was proposed that the industry adopt a philosophy that encapsulate six key components: measuring, understanding and learning; the set of experiments identified; data generated; data ingested and processed; analysis of the data context; the training and testing of the models and new updated insights. He predicted that this would assist in the understanding of experiments and their success criteria; draw attention to metadata quality; understand objectives and flexible analytical pipelines; apply the best active and machine learning methods to scientifically challenge and interpret output to drive the delivery of new scientific insights.

Specialist expertise in data manipulation has traditional only been available in academic institutions and finance houses are now not only emerging through a new generation of start-up companies providing services to the pharmaceutical industry but also through huge data-driven organizations like Google, Apple, and Amazon, who are beginning to show an interest in the healthcare sector. It was noted that those companies that can harness such knowledge are poised to make significant break-throughs, not only in terms of pipelines and processes, but also in the way that the pharma businesses will control, shape, and steer data through their entire organization.

Classic drug discovery pipelines of the future were suggested to become more agile if data driven, active and machine learning applications were implemented from the outset and maintained through to the final stages of the development process. He described how data can be used to predict efficacy, toxicology, and manufacturing issues in the transition from lead identification to candidate optimization stage and eventually through to target validation. He predicted that this would have a ripple effect on the other transition stages where integrated data could predict potential biomarkers, disease progression, and treatment response. Effectively, data can be enriched by breaking down silos within and between the teams and by differentiating “positive” and “negative” data using labels. Introducing appropriate anonymization allows in-house safety databases on subjects exposed to candidate molecules to be enriched from external resources, e.g., genomics England. Metadata could be used to standardize vocabularies and units across clinical data sets which would aid with code versioning, testing and validation processes to bridge communicators.

The Aigenpulse platform was introduced as one such solution to organizing scientific data efficiently; where deployment of their experimental suite is expected to promote re-usability, increase efficiency, and drive data quality.

The presentation was concluded with the concept that only by becoming part of the data revolution will life science organizations be able to remain dynamic, innovative, and relevant. In the coming decade, winners in the race for total digital transformation will be those that adapt agile and integrated approaches to data management and manipulation.

## Conclusion

The 2019 AHPPI meeting focused on a selection of the changes in early phase drug development that have occurred in the last 4 decades, current innovations in pharmaceutical medicine and the future challenges facing the pharmaceutical industry. A wide range of exciting topics were presented and discussed including advances in and the future of biologics and how the industry and regulatory agencies are adapting to novel trial designs. The event included a lively discussion over the future of placebo subjects in Phase I trials and the keynote address on addressing the issue of delivering successful change. Further developments that were discussed included the biopharmaceutical impact of the delivery of molecular drugs via non-oral routes of administration as well as the simulation and *in silico* environment in Phase I trials and data driven transformation in drug development. In closing the meeting, the AHPPI Chairman, Dr. Tim Hardman, summarized how the topics discussed demonstrated how the pharmaceutical industry is quickly changing and the pharmaceutical industry in the next 10 years will likely be very different.

## Author Contributions

All the authors were responsible for determining the academic content of the AHPPI meeting, recruiting the various presenters, and noting the information presented at the meeting. All authors worked with the rest of the AHPPI Committee to compile the various presentations, write, and review as well as approve the article. Presentations given by invited speakers are published on https://www.bps.ac.uk/news-events/events/2019/ahppi-annualmeeting. All authors listed have made substantial, direct, and intellectual contribution to the work and approved it for publication.

## Conflict of Interest

MA was employed by the company Parexel International. JB was employed by the company Covance. AC was employed by the company Celerion. AK was employed by the company Simbec-Orion Group. SM was employed by the company Quotient Sciences. UL and JT were employed by the company Richmond Pharmacology Ltd. SS was employed by the company Alwyn Consulting. SK was employed by the company Niche Science & Technology. TCH is the Managing Director of the company Niche Science & Technology.

The remaining authors declare that the research was conducted in the absence of any commercial or financial relationships that could be construed as a potential conflict of interest.
